# Impedance Spectroscopy as a Tool for Monitoring Performance in 3D Models of Epithelial Tissues

**DOI:** 10.3389/fbioe.2019.00474

**Published:** 2020-01-24

**Authors:** Tatiana Gerasimenko, Sergey Nikulin, Galina Zakharova, Andrey Poloznikov, Vladimir Petrov, Ancha Baranova, Alexander Tonevitsky

**Affiliations:** ^1^Scientific Research Centre Bioclinicum, Moscow, Russia; ^2^Laboratory of Microphysiological Systems, School of Biomedicine, Far Eastern Federal University, Vladivostok, Russia; ^3^Laboratory of Molecular Oncoendocrinology, Endocrinology Research Centre, Moscow, Russia; ^4^Department of Translational Oncology, National Medical Research Radiological Center of the Ministry of Health of the Russian Federation, Obninsk, Russia; ^5^Department of Development and Research of Micro- and Nanosystems, Institute of Nanotechnologies of Microelectronics RAS, Moscow, Russia; ^6^School of Systems Biology, George Mason University, Fairfax, VA, United States; ^7^Laboratory of Molecular Genetics, Moscow Institute of Physics and Technology, Dolgoprudny, Russia; ^8^Laboratory of Functional Genomics, “Research Centre for Medical Genetics”, Moscow, Russia; ^9^Faculty of Biology and Biotechnologies, Higher School of Economics, Moscow, Russia; ^10^Laboratory of Microfluidic Technologies for Biomedicine, Shemyakin-Ovchinnikov Institute of Bioorganic Chemistry RAS, Moscow, Russia; ^11^art photonics GmbH, Berlin, Germany

**Keywords:** impedance spectroscopy, TEER, epithelium, barrier tissues, 3D cell culture models, organs-on-a-chip, microfluidic devices, label-free monitoring

## Abstract

In contrast to traditional 2D cell cultures, both 3D models and organ-on-a-chip devices allow the study of the physiological responses of human cells. These models reconstruct human tissues in conditions closely resembling the body. Translation of these techniques into practice is hindered by associated labor costs, a need which may be remedied by automation. Impedance spectroscopy (IS) is a promising, automation-compatible label-free technology allowing to carry out a wide range of measurements both in real-time and as endpoints. IS has been applied to both the barrier cultures and the 3D constructs. Here we provide an overview of the impedance-based analysis in different setups and discuss its utility for organ-on-a-chip devices. Most attractive features of impedance-based assays are their compatibility with high-throughput format and supports for the measurements in real time with high temporal resolution, which allow tracing of the kinetics. As of now, IS-based techniques are not free of limitations, including imperfect understanding of the parameters that have their effects on the impedance, especially in 3D cell models, and relatively high cost of the consumables. Moreover, as the theory of IS stems from electromagnetic theory and is quite complex, work on popularization and explanation of the method for experimental biologists is required. It is expected that overcoming these limitations will lead to eventual establishing IS based systems as a standard for automated management of cell-based experiments in both academic and industry environments.

## Introduction

*In vitro* cell models are indispensable as the tools of modern biology and medicine; these models are widely used in studies of molecular pathogenesis and metabolism of bioactive compounds (Astashkina et al., 2012; Caicedo-Carvajal et al., 2012; Marx et al., 2016). Nowadays, cell-based models have gained their popularity as a replacement for laboratory animals, especially in the area of drug discovery, where these models improved productivity in a cost efficient way (Doke and Dhawale, 2015; Poloznikov et al., 2018). Recently, traditional 2D cell culture models have evolved into 3D tissue-engineered scaffolds, organ-on-a-chip platforms and organoid test beds (Marx et al., 2016; Dehne et al., 2017; Maschmeyer et al., 2017; Spielmann and Marx, 2017; Torras et al., 2018). These physiologically relevant systems allow experiments with various human cells in conditions resembling the ones found in the human body. It is important to recognize that human-based 3D models and organ-on-a-chip devices provide several advantages over animal testing as human biological processes differ from those in a typical laboratory animal, the majority of which are rodents. Moreover, these models open up the possibilities for personalized testing.

Currently, monitoring of a cell's state mainly depends on assaying various endpoints require introducing one or another type of label. Endpoint assessment techniques are laborious, expensive and often disruptive, as they require a portion of biological material collected before each test. Hence, the recent advent of real-time label-free assays is not surprising (Limame et al., 2012; Single et al., 2015). Non-invasive, label-free longitudinal monitoring of cell states is a key component for the development of automated microphysiological systems slated to be eventually adopted by industry.

Impedance spectroscopy (IS) is a label-free technique suitable for quantification of cell properties in real time. One of the common applications of impedance measurement is quality control (QC) for *in vitro* models of barrier tissues. Being compatible with a variety of culture formats, IS has already found its way into organs-on-a-chip devices. Here we review applications of impedance spectroscopy with special attention to 3D cell culture formats and 3D structures formed by barrier tissues.

## Basic Theory of Impedance Spectroscopy

Impedance is a generalization of the concept of “resistance” in the case of an alternating current. By definition, impedance is a proportionality factor between the alternating voltage *V* with frequency *f* applied to the system under investigation and the electric current *I* flowing through it:

$$\begin{array}{l} Z\left(f\right)=\frac{V\left(f\right)}{I\left(f\right)} \end{array}$$

Since the conventional description of alternating current and voltage involves complex numbers, impedance is also a complex quantity, which may be written as follows (Cartesian form):

$$\begin{array}{l} Z=\text{Re}\left[Z\;\right]+j\text{Im}\left[Z\right], \end{array}$$

where Re[*Z*] and Im[*Z*] are the real and imaginary parts, ***j*** is the imaginary unit (*j*^2^ = −1). The equivalent polar form can also be used:

$$\begin{array}{l} Z=|Z|e^{j\phi } \end{array}$$

**Figure 1:**
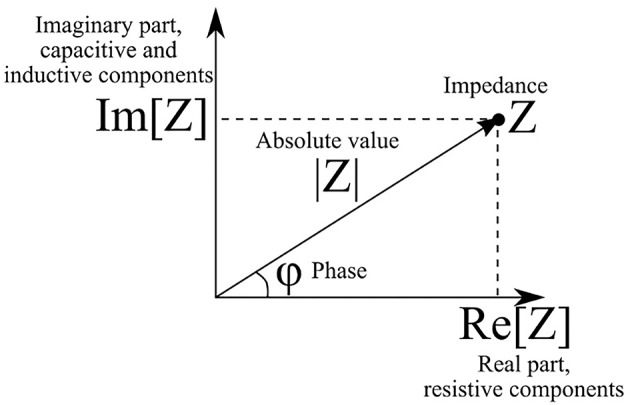
Phasor diagram of complex impedance (illustrates the relationship between Cartesian and polar representations of complex number).

The absolute value *|Z|* of impedance and phase shift between current and voltage φ are related to the real and imaginary parts of impedance as follows (Figure 1):

$$\begin{array}{l} |Z|=\sqrt{{\text{Re}\left[Z\right]}^{2}+{\text{Im}\left[Z\right]}^{2}}\\ \varphi =\text{arctan }\frac{\text{Im}\left[Z\right]}{\text{Re}\left[Z\right]} \end{array}$$

**Figure 2:**
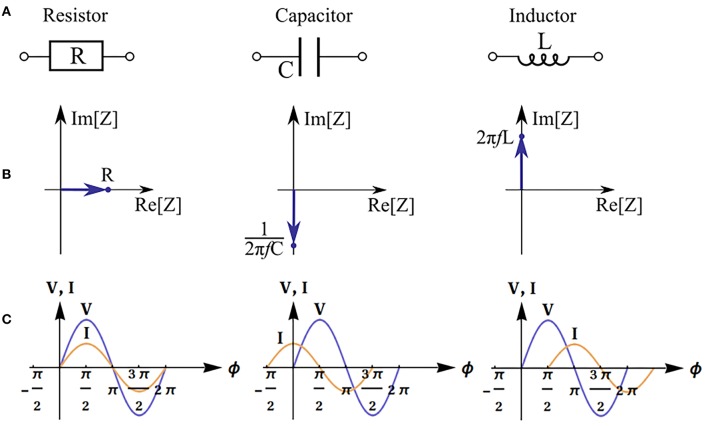
Impedance of basic equivalent circuit components: resistor, capacitor and inductor. Electronic schematic symbols **(A)**, phasor diagrams and impedance values (f is the frequency) **(B)**, and illustration of phase shift between voltage (*V*) and current (*I*) (φ is the phase) **(C)**.

The real part of impedance is responsible for dissipation of energy in the system (active resistance). The imaginary part describes electrical capacitance and induction of the system. When the imaginary part is not equal to zero, there is a phase shift between current and voltage (Figure 2). The magnitude of the shift also depends on the real part of impedance.

**Figure 3:**
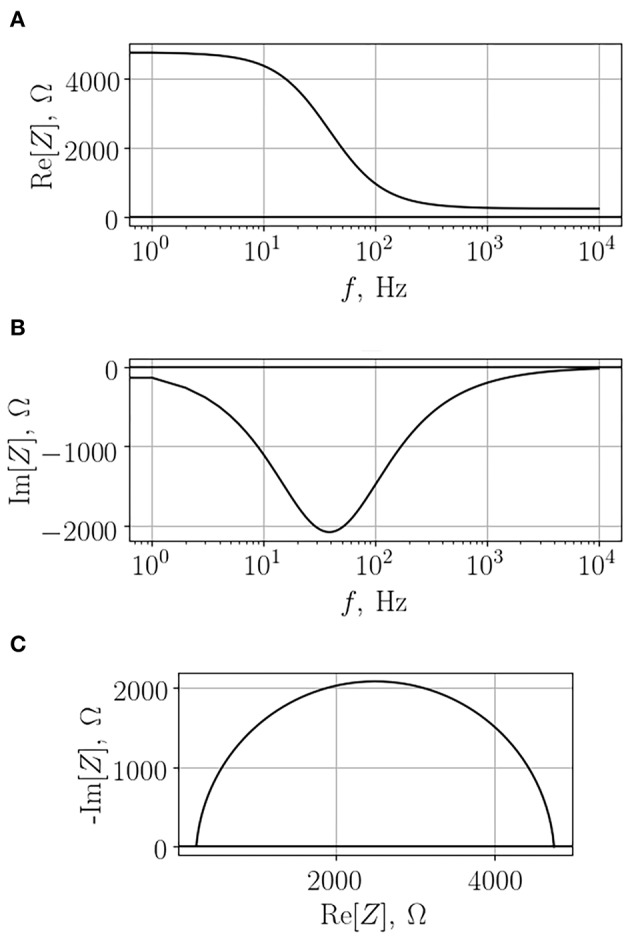
Typical impedance spectrum of a cell monolayer grown on a semi-permeable membrane. The real part **(A)**, the imaginary part **(B)**, and the Nyquist diagram **(C)**.

Impedance depends on the frequency of the applied voltage; therefore, in order to obtain comprehensive information on the system, one has to scan a range of frequencies in order to generate so-called impedance spectrum. A typical example of such a spectrum of a cell monolayer growing on a semipermeable membrane is depicted in Figure 3. As one can see, in this case both real and imaginary parts of the impedance change with the change of frequency. Importantly, the dependency of imaginary part on frequency is not monotonous. Analysis of the obtained impedance spectra often includes construction of Nyquist plot that reflects the dependence of -Im[*Z*] on Re[*Z*]. This type of graphs facilitates interpretation of resultant data, each configurations of the studied model systems will be recognized by a characteristic shape of observed impedance spectrum.

In the simplest case, a cell may be considered as a plasma membrane shell filled with cytoplasm (Morgan et al., 2007). Both the conductivity σ_ct_ and permittivity ε_ct_ of a living cell are assumed to be of the same order of magnitude as the properties of the surrounding extracellular liquid. At the same time, its membrane is considered as an insulator with permittivity ε_m_ << ε_ct_ and conductivity σ_m_ << σ_ct_. As the membrane of a dead cell becomes perforated, its ability to obstruct the traffic of ions would be lost. Therefore, its conductivity approaches that of the extracellular liquid (Fricke, 1924; Lvovich, 2012; Castellví, 2014).

One may examine directly the dependency of complex permittivity instead of impedance on frequency. Complex permittivity is a value that integrates permittivity and conductivity into a single value in the following manner:

$$\begin{array}{l} \varepsilon \left(f\right)=\varepsilon^{\prime }\left(f\right)\;+\;j\varepsilon^{\prime \prime }\left(f\right)=\varepsilon_{r}\left(f\;\right)\varepsilon_{0}\;+\;j\frac{\sigma \left(f\right)}{2\pi f}, \end{array}$$

where ε_r_ is a relative permittivity and ε_0_ is a dielectric constant. This approach, which is widely used to study electrical properties of various suspensions, relies on models that consider the dispersion of electromagnetic waves in a continuous medium (Schwan, 1994; Morgan et al., 2007; Sun et al., 2007). These models provide an insight into the physics of the system under study. A study of distributed parameters such as conductivity and permittivity requires quite complex mathematical apparatus; therefore, lumped parameter models are more attractive. As an example, one may consider conductivity as a resistance *R*:

$$\begin{array}{l} \text{Re}\left[Z\right]=R,\text{Im}\left[Z\right]=0 \end{array}$$

On the other hand, permittivity can be considered as a capacitor *C*:

$$\begin{array}{l} \text{Re}\left[Z\right]=0,\text{Im}\left[Z\right]=\frac{1}{j2\pi fC} \end{array}$$

**Figure 4:**
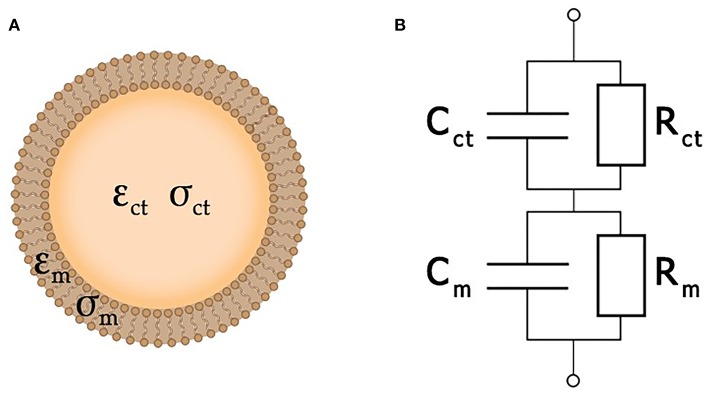
A simplified cell structure **(A)** and its equivalent circuit **(B)**.

Here, the single-shell model (Xu et al., 2016) is used to describe each cell as an equivalent circuit (Figure 4), where *C*_*m*_ and *R*_*m*_ correspond to the cellular membrane, while *C*_*ct*_ and *R*_*ct*_ correspond to the cytoplasm. A typical value of membrane conductance per unit area is about 0.3 mS/cm^2^, its specific capacitance is about 1 μF/cm^2^, and the cytoplasm conductance σ_*ct*_ is about 0.005 S/cm^2^ (Asami et al., 1996). The value of *R*_*m*_ is usually much greater than *R*_*ct*_, while *C*_*ct*_ is much smaller than *C*_*m*_. Because of that, analysis of the impedance is amenable to simplification by neglecting *C*_*ct*_ and *R*_*m*_. At very high frequencies (*f* > 100 MHz), the cell membrane capacitance *C*_*ct*_ is effectively short-circuited, and the impedance is then determined by the cytoplasm resistance (Sun et al., 2008). More detailed information about single cell equivalent circuits can be found in a review (Xu et al., 2016). Yet another degree of simplification may be achieved by the replacement of the entire system under study by an equivalent circuit. In this case, the resulting impedance is expressed analytically in terms of the parameters of each separate element of the equivalent circuit, which, in turn, are estimated by fitting experimental data.

At the same time, the method of equivalent circuits implies a significant simplification of the processes that take place in real biological objects. This simplification is primarily due to the assumption of ideal electrical characteristics. The majority of impedance spectra may be described by more than one equivalent circuit. Selection of this circuit relies on initial judgment calls made by the researcher, which reflect his understanding of underlying biological processes. When the choice of the equivalent circuit is far from being optimal, the results may in fact end up incorrectly interpreted as well. Particular care is required when equivalent circuits incorporate large numbers of elements. In these cases, the relevance of a given circuit configuration is difficult to establish, even though excellent fitting of the data can be achieved (McAdams and Jossinet, 1996).

## Practical Aspects of Impedance Measurements in Cell Culture Models

The impedance measurement procedure should not significantly affect the state of the cells. This imposes certain restrictions on the characteristics of the electromagnetic fields used. In particular, transmembrane potential should remain significantly lower than the threshold value for membrane electroporation (250–350 mV). Typically, this restriction is not a problem, since such values of the membrane potential are usually achieved by external fields with a strength of the order of 1 kV/cm. It should be noted, however, that muscle and nerve cell membranes can be damaged with electrical fields as small as 60 V/cm (Lee, 2005). In addition, both relatively weak constant electric fields with field strengths about 0.1–10 V/cm and electromagnetic fields with extremely low frequency are capable of affecting cytoskeleton and cell shape, influencing migration, proliferation, and differentiation of at least some types of human cells (Funk and Monsees, 2006). Moreover, electrical fields of 1–10 V/cm applied to a cell of 10 μm in radius can change the membrane potential by as much as 1.5–15 mV, which, in turn, may alter the activity of some membrane channels (Mycielska and Djamgoz, 2004; Funk and Monsees, 2006; Taghian et al., 2015). Therefore, one has to ensure that applied voltage (or current) produces a field with the strength not exceeding specified values.

The choice of such specific values of applied voltage or current strongly depends on the spatial arrangement of electrodes and cells, resistance of culture medium and number of cells. As a rule of thumb, investigations of suspensions and *in vitro* models of barrier tissues call for relatively small currents of about tens of μA (Gitter et al., 2000; Krug et al., 2009). In the case of 3D cultures, voltages of 10–100 mV may be applicable (Thielecke et al., 2001b; Canali et al., 2015a). On the other hand, in microfluidic devices, due to a significant voltage drop observed in microchannels, the potential difference across the electrodes can reach 0.1–0.5 V (Gawad et al., 2001; van der Helm et al., 2016).

**Figure 5:**
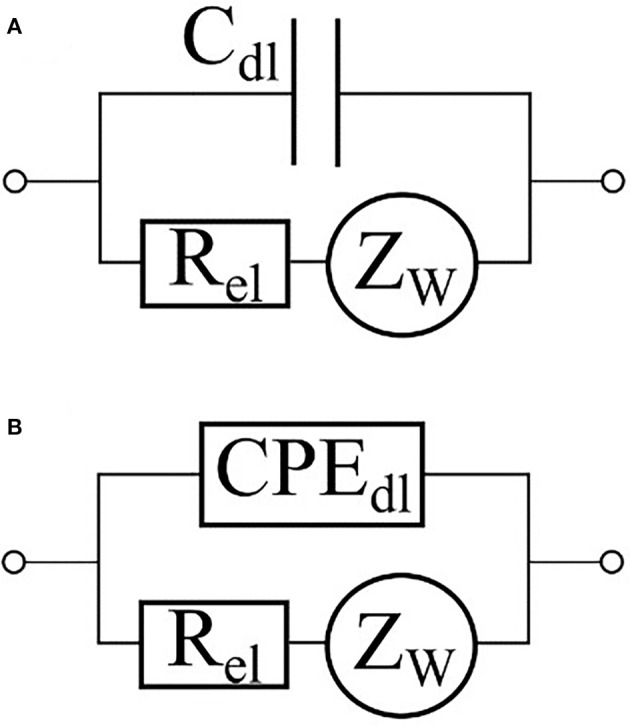
Equivalent circuits of an electrode-electrolyte interface. The double-layer capacity is represented either by a capacitor **(A)** or by a constant phase element **(B)**.

In a majority of applications of impedance spectroscopy to living matter, electrodes are placed in direct contact with culturing medium. In this case the interface between the medium and the electrode should be included into complete equivalent circuit of the system under study. The interactions between electrode and medium are usually described by an equivalent circuit shown at Figure 5 (Grafov and Ukshe, 1973) where *R*_*el*_ is a charge transfer resistance, $Z_{W}=\frac{W_{F}}{\sqrt{2\pi f}}\;\left(1-j\right)$ is an impedance of Warburg element describing a Gouy-Chapman diffusive layer, and W_F_ is the Warburg constant. The Helmholtz double-layer is usually modeled by a capacitor *C*_*dl*_. (Figure 5A); however, in some cases, a constant phase element (CPE) $Z_{CPE}=\frac{A}{{\left(2\pi f\right)}^{\alpha }}\left(\text{cos}\frac{\pi \alpha }{2}-j\text{sin}\frac{\pi \alpha }{2}\right)$ with *A* > 0 and 0 ≤ α ≤ 1 as constant values may be used instead (Figure 5B) (Moulton et al., 2004; Chang et al., 2007; Yang et al., 2008). A review of Chassagne et al. (2016) delves into a detailed theory of electrode polarization processes as well as ways to compensate them. In particular, a four-electrode scheme helps to eliminate the influence of interface between the electrode and the medium on data output (Amini et al., 2018).

Both the shape and the size of an electrode are of extreme importance. Generally speaking, each experimental setting requires electrodes of specific shape and size, with particular designs being guided by theoretical calculations (Franks et al., 2005; Abdur Rahman et al., 2007; Alexander et al., 2010; MacKay et al., 2015). In the case of rod-shaped electrodes, the results of the measurements may be influenced by the relative positioning of electrodes and the distance to the studied monolayer (Srinivasan et al., 2015). Moreover, since Helmholtz double layer impedance is inversely proportional to the surface area, in the case of microelectrodes, this effect may lead to very large impedances, particularly at low frequencies (Alexander et al., 2019). One of the possibilities to overcome this problem is to add 3D micro- or nanostructures on top of the sensing electrodes as it is described in details (Decker et al., 2018).

Therefore, in order to decrease the impact of this interface, the electrode area should be as large as possible. Another important factor which decreases the accuracy of the measurement is the background noise caused by chemical processes taking place on the surface of the electrode. This noise is especially important when the magnitude of the signal is low. Increasing the surface area of the electrodes allows electrode-electrolyte interface noise to be lowered (Huigen et al., 2002).

As impedance-measuring electrodes function in close proximity to the living cells, the electrode material should not cause any toxic effect and remain chemically and physically stable across the experiment. Gold, platinum, palladium, and titanium are the materials of choice (Hoffmann et al., 2006; Riistama and Lekkala, 2006; Pliquett et al., 2010; Howlader et al., 2013), with some researchers experimenting with indium tin oxide, nickel, ultra-nanocrystalline diamond, and electrolyte solutions (Xu et al., 2016). Silver chloride electrodes have become a primary component of many electrochemical chambers due to their low cost and stable potential (Shinwari et al., 2010). However, the contact with biological media greatly enhances erosion of such electrodes, causing the loss of AgCl coating, leading to a change of electrode potential and marked cytotoxicity. Even with the development of stabilizing coatings (Kaji et al., 1995; Polk et al., 2006; Riistama and Lekkala, 2006; Shinwari et al., 2010), these electrodes are less biocompatible than the ones based on gold, platinum and titanium. However, it is still true to say that silver chloride electrodes are useful for short time measurements. For example, STX-electrodes compatible with EVOM^2^ (World Precision Instruments) are made from silver/silver chloride. It is also worth noting that some powder metallurgy-produced titanium alloys containing Mo, Nb, or Si show a certain degree of cytotoxicity (Li et al., 2010).

## Investigations of Barrier Functions

**Figure 6:**
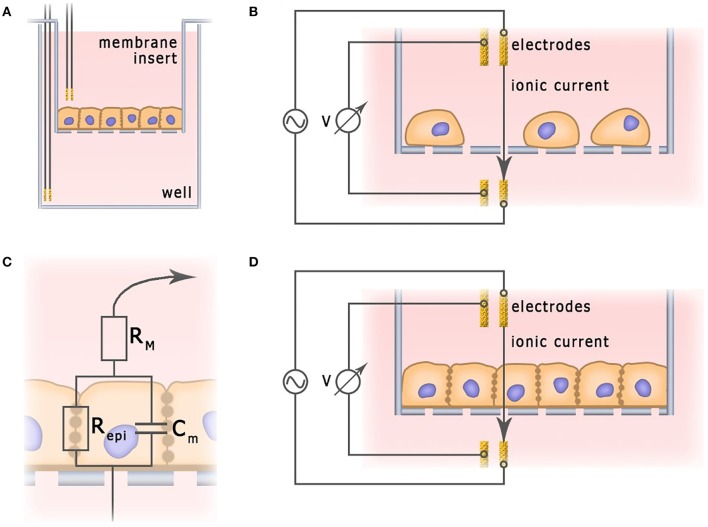
Application of impedance spectroscopy for the measurement of barrier functions. **(A)** Typical electrochemical cell of trans-epithelial resistance measurement. **(B)** Current flow through undifferentiated low density cell culture. **(C)** Simplified equivalent circuit of cellular monolayer. **(D)** Current flow through fully differentiated cell culture.

A characteristic feature of epithelial and endothelial cells is their ability to form tight junctions. Monolayers of tightly connected cells create a selectively permeable interface between apical and basal compartments, thus controlling diffusion and transport of chemical substances (Benson et al., 2013). The integrity of this barrier is vital for normal physiological functionality of the tissue. In order to deliver therapeutic agents to the targeted organs, this barrier has to be penetrated, but not destroyed. In studies of the permeability of epithelial and endothelial barriers, cells are often grown on semipermeable membranes (Figure 6), where the integrity of the cell monolayers could be controlled non-invasively, by measuring trans-epithelial resistance (TEER) (Samatov et al., 2015; Srinivasan et al., 2015). TEER measurements are commonly utilized for monitoring of conventional 2D cultures of epithelial cell lines such as Caco-2 and HT-29 (Hilgendorf et al., 2000). Their applicability for *in vitro* models of barrier tissues derived from primary 3D organoids has been discussed as well (Moon et al., 2014).

In a typical TEER unit, cells grow on a semipermeable membrane with the electrodes placed in apical and basal compartments separated by monolayer. In a unit depicted at Figure 6, two electrodes provide current, and two other electrodes measure voltage. In some other designs, employing only two electrodes, one of them is placed in the basal and another one—in the apical compartment (Bragós et al., 2006; Yufera and Rueda, 2008). In theory, monolayer integrity could be probed with the direct current, but the polarization of electrodes and the monolayer itself provided by constant electric field calls for use of an alternating current at a low frequency (about 10 Hz). For example, EVOM^2^ (World Precision Instruments) and Millicell ERS-2 (EMD Millipore Corporation) devices, which can be used in conjunction with a chopstick silver/silver chloride electrode, operate at a single frequency of 12.5 Hz and current 10 μA, providing information on TEER. EVOM^2^ is also compatible with EndOhm chamber (World Precision Instruments) which contains a pair of concentric electrodes, including a voltage-sensing silver/silver chloride pellet in the center and an annular current electrode around it. Symmetrical arrangement allows to generate uniform current density across the membrane, and, therefore, preferable for chopstick electrodes (Srinivasan et al., 2015).

**Figure 7:**
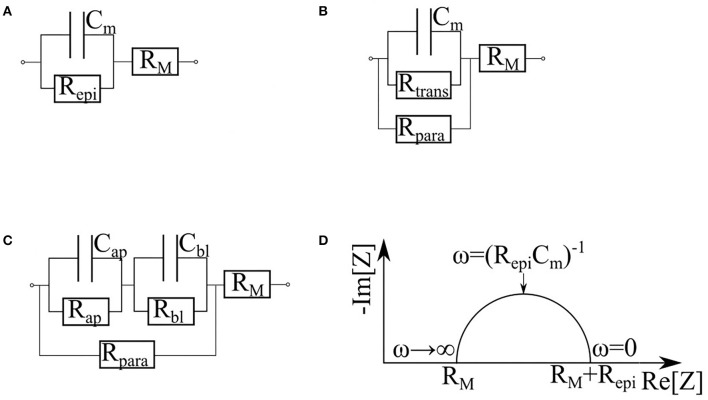
Various equivalent circuits representing the cell, based on (Krug et al., 2009). R_M_ is the sum of subepithelial resistance, medium resistance and semipermeable membrane resistance. **(A)** The simplest equivalent circuit, which does not differentiate cytoplasm and tight junctions. R_epi_ is the trans-epithelial resistance. C_m_ is the membrane capacitance. **(B)** Circuit dividing the current flowing through the cells (R_trans_) and through the tight junctions (R_para_). **(C)** Circuit which represents contribution of apical and basal membranes separately (R_ap_, C_ap_ and R_bl_, C_bl_ correspond to the apical and basal membrane respectively). **(D)** Nyquist plot of cell monolayer.

Replacement of measurements at a single frequency with impedance spectroscopy provides an opportunity to collect additional information describing various properties of monolayer in question. Depending on particular equivalence circuit, one or another set of parameters may be collected. In barrier cultures, both membrane resistance and cytoplasm capacitance are negligible (Figure 7). In the simplest case (Figure 7A) the monolayer is represented as a resistor and a capacitor in parallel. The capacitor *C*_m_ corresponds to the membrane contribution whereas the resistor *R*_epi_ reflects the transport through the cell and tight junctions. For this circuit *C*_m_, both *R*_epi_ and *R*_M_ can be determined directly from the Nyquist plot of the impedance spectrum (Figure 7D) (Schifferdecker and Frömter, 1978; Fromm et al., 1985). This model is especially useful when the loss of barrier function occurs simultaneously with the increase of subepithelial resistance, for instance, in course of intestinal tissue inflammation (Bürgel et al., 2002; Zeissig et al., 2007). It is worth to note that fitting of experimental impedance spectra may be improved by replacing the capacitor *C*_m_ with a constant phase element (CPE) described by parameters *A* and α (Cole, 1932; Grimnes and Martinsen, 2005, 2015; Lazarevi and Caji, 2015).

When equivalent circuits become more elaborate (Figure 7B), a direct estimation of all unknown parameters from the impedance spectrum becomes impossible, thus, requiring additional measurements. For example, in the study of paracellular transport modulated by addition of egtazic acid (EGTA), *R*_trans_ and *R*_para_ parameters were sorted out with an aid of complementary measurements of a fluorescein flux (Krug et al., 2009). The contributions of the apical and basolateral membranes (Figure 7C) can be dissected if their time constants (τ_*ap*_ = *R*_*ap*_*C*_*ap*_ and τ_*bl*_ = *R*_*bl*_*C*_*bl*_) differ substantially (Sackin and Palmer, 2013). As an example, an exposure to nystatin has selectively shown an increase in Na+, K+, and Cl– conductivity of an apical membrane by several orders of magnitude (Lewis, 1977; Wills et al., 1979), therefore decreasing its time constant. Another example of similarly designed experiment would be an activation of cAMP-dependent channels of apical membranes by forskolin (Krug et al., 2009). The influence of the latter on the impedance spectra is described in Păunescu and Helman (2001). A more complex apical/basal discrimination technique requires direct insertion of electrodes into the cells (Frömter and Diamond, 1972; Schifferdecker and Frömter, 1978; Kottra and Frömter, 1984). To collect detailed information on the local conditions within particular places of the monolayer, microscopic scanning electrodes may be placed a micrometer away from its apical surface (Cereijido et al., 1980; Gitter et al., 1997, 2000). This technique allows quantifying local variations in the current density, which reflect the state of the monolayer at each particular point while discriminating electrical properties of the cells and the tight junctions.

To date, several IS systems were designed and implemented in investigations of barrier cell cultures. For example, cellZscope systems (nanoAnalytics GmbH) relies on a single-piece stainless steel bottom electrode “pots” used both as reservoirs for culture medium, and as a support for membrane inserts (Veltman et al., 2012; Valere et al., 2015). A lid of this “pot” serves as upper electrode, which generates uniform electric field across the membrane inserts. The device operates at frequency range between 1 Hz and 100 kHz, and provides information on TEER, medium resistance and capacitance of cell monolayer. Another IS systems, which is compatible with standard commercially available electrodes for 96-well membrane inserts, was recently developed by our team (Nikulin et al., 2019a,b).

Usually the same electrode layouts as for TEER measurements are used for impedance profiling. However, high cell resistance at low frequencies may result in low measured currents and high levels of noise. To overcome this problem, the laboratory of Dr. Owens (Jimison et al., 2012; Ramuz et al., 2014; Rivnay et al., 2015) replaced conventional electrodes with organic electrochemical transistors (OECT), and used them to measure the integrity of the barrier tissue. In the OECT, there is no direct measurement of the resistance across the cell monolayer. The drain current depends on the speed at which the transistor reaches steady state. Utilization of both the gate and drain current of an OECT allowed the authors to perform frequency-dependent impedance measurements over a broad range of frequencies while collecting high quality data at low frequencies.

When integrated into microfluidic organ-on-a-chip devices, impedance measuring electrodes allow long-term monitoring of the cultured cells in a controlled environment (Douville et al., 2010; Booth and Kim, 2012; Griep et al., 2013; Huang et al., 2014; Walter et al., 2016). The size constraint, which is common with respect to microfluidic platforms, dictates the placement of electrodes in close proximity to the cells. When the electrodes are located too close to the cell monolayer, the resultant electric field is far from being uniform. Therefore, the impact of each cell at the total TEER values depends on the position of a given cell along the electrodes, and overall cell confluence. As it has been demonstrated by Odijk et al. (2015), in microfluidic chips, TEER values obtained for the same type of cells may vary greatly, and are often different from those measured in Transwell systems.

The subsequent electrical impedance simulation method proposed by Odijk et al. (2015) was developed by the same scientific group (van der Helm et al., 2019) to normalize the cell layer resistance to TEER. The microfluidic chip was modeled, as a distributed electrical network comprised of different of elements, corresponding to culture medium, electrode, cell layer, and semipermeable membrane. The epithelial resistance derived from simulated impedance spectra was plotted against the input TEER, resulting in a calibration curve, giving the possibility of obtaining the TEER values from experimentally determined resistance. Yeste et al. (2016) suggested that TEER values should be calculated using a so-called geometric correction factor (GCF):

$$\begin{array}{l} GCF=\frac{TEER_{t}}{TEER_{s}} \end{array}$$

where *TEER*_s_ is the *TEER* value obtained from mathematical simulation and *TEER*_*t*_ is used as a parameter for the electrical conductivity of the small volume in the middle of the two chambers, which represents a cell layer.

In our own studies, an organ-on-a-chip platform called “Homunculus” was recently upgraded to include an impedance spectroscopy system for real-time monitoring of the barrier function (Sakharov et al., 2017). In this device, prefabricated multi-well microfluidic chips for the co-culture of intestinal and placental barrier tissues with non-barrier cells such as hepatocytes include electrodes (Marx et al., 2016; Poloznikov et al., 2018). Utilization of this type of chip greatly reduces the time and effort of impedance measurements, providing integrative estimation of intestinal permeability while quantifying the rates of biotransformation and profiling the toxicity of tested compounds. Another organ-on-a-chip combination of the TEER impedance measurement system with microelectrode array (MEA) aimed to model the endothelialized myocardium (Maoz et al., 2017). In this system a microfluidic chip, porous PET membrane separates two microchannels. An apical chamber holds endothelial cells, while cardiomyocytes populate a basal channel. The dual sensor system (TEER-MEA) allows monitoring of the endothelial barrier function and electrical activity of the cardiomyocytes within the same device.

**Figure 8:**
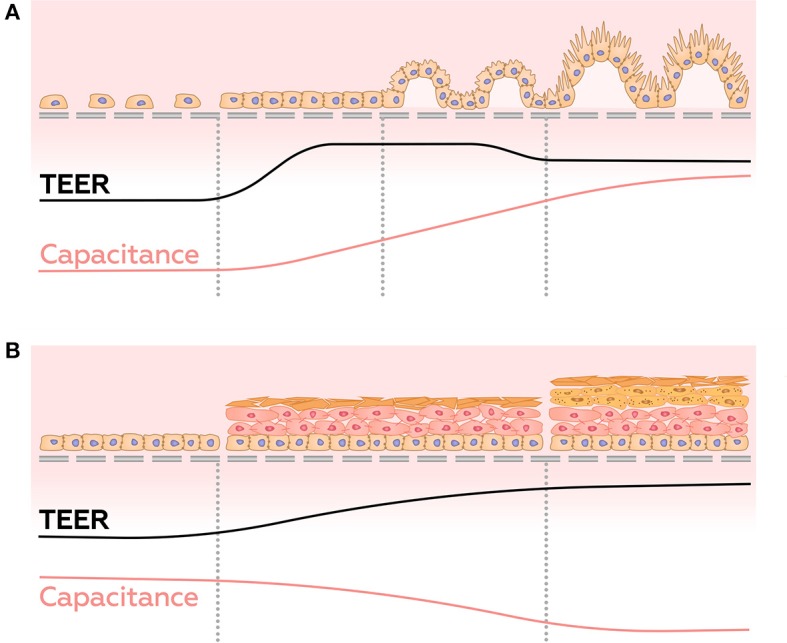
Dynamics of TEER and capacitance changes during growth and differentiation of gut epithelial cells **(A)**, and during the development of reconstructed epidermis **(B)**.

In contrast to conventional TEER measurements, impedance spectroscopy provides a window into overall well-being and the stage of differentiation acquired by 3D structures formed by barrier cells. For example, it has been shown that in the first few days after the seeding of cells, the TEER values peak, then decrease along with differentiation (Henry et al., 2017; Nikulin et al., 2018), possibly due to the appearance of villi and microvilli on the surface of the membrane (Geens and Niewold, 2011; van der Helm et al., 2019). On the other hand, the capacitance increases continuously during cell growth, due to the gradual increase of the area of the cell membrane (Figure 8A) (Henry et al., 2017; Nikulin et al., 2018; van der Helm, 2018; van der Helm et al., 2019). Thus, simultaneous measuring of TEER and electrical capacitance may adequately report about the degree of differentiation in culture.

Some cultures of epithelial barrier cells are known to form 3D multilayer structures, which may be represented by several parallel resistor-capacitor (or CPE)-circuits. Analysis of these circuits may provide information about layers' number and/or their structure. This approach was employed in a study of maturation of a reconstructed human epidermis (RHEs) (Groeber et al., 2015). Before developing any architecture, RHEs could be described as a single monolayer. In the interim phase, cells pile up, and corneous layer begin to form, building up dual layer architecture. In the late phase, the corneous layer has strengthened, and its electrical properties become dominant. At this stage, the system returns to a single monolayer architecture. Throughout RHE maturation, these measurements demonstrate an increase in transepitelial resistance and a decrease in CPE parameter *A*, whereas parameter α, which interpreted as so-called “ideality” of a capacitor, undergo slightly decrease (Figure 8B). Sensitivity of the system described above allows distinguishing effects of strong skin irritants and non-irritants. When similar methodology was employed to investigate formation of multilayers of choriocarcinoma BeWo b30 (Nikulin et al., 2019b), observed shifts of the semi-circle center of Nyquist plot to the right and down allowed making conclusions about amounts of accumulated cell layers (Figure 7D).

The spectrum of barrier interface models amenable to TEER monitoring were recently expanded to the blood-brain barrier (BBB), which was reliably reconstituted by co-culturing human induced pluripotent stem cell (hiPSC)-derived brain microvascular endothelial cells with membrane-separated astrocyte-laden 3D hydrogel embed in a BBB-on-a-chip device that supports flow. In this model, apical addition of TGF-β1 led to the reduction of TEER and activation of astrocytes (Motallebnejad et al., 2019). There is a hope that this TEER-enabled BBB-emulating device could be used as BBB disruption model and find its use in drug screening settings.

## 3D Cell Culture

Initially, the developers of impedance spectroscopic techniques aimed at studying of cells maintained either in suspensions or in conventional 2D cultures. The growing trend of 3D culturing, which provides the cells with a physiologically relevant microenvironment (Rivnay et al., 2015), has led to a number of attempts to adapt IS to 3D. In typical laboratory settings, performing *in vitro* tests in 3D cell models is more difficult and time-consuming than in 2D. In particular, scattering effects observed in 3D constructs hampers the use of conventional optical techniques due to their thickness. Impedance spectroscopy allows relatively non-invasive, real-time glimpse into well-being of 3D cell cultures in lieu of optical microscopy or use of destructive methods.

The most widely used 3D cell culture technique produces so-called spheroids (Zanoni et al., 2016), which are formed by suspended cells either spontaneously or under the influence of various external factors. To date, several published works have employed impedance spectroscopy for the measurement of various properties of spheroids. For example, Thielecke et al. (2001a) used circular planar electrodes both to investigate the effect of bioactive substances on multicellular spheroids, and to profile the impedance produced by placing the electrodes at various distances from the spheroids. As a result, an optimal electrode/spheroid-interface for sensing the effects of drugs has been designed.

A microcavity array (MCA) biosensor chip was subsequently developed (Kloß et al., 2008a,b; Eichler et al., 2015). The chip consists of several square microcavities with rectangular gold electrodes. The impedance measured between any pair of electrodes increases if a microcavity contains a spheroid. An MCA chip can be used to assess cytotoxic effects of chemotherapeutic drugs. The data generated by impedimetric monitoring of the chemotherapeutic toxicity generally agree with the results of conventional cytotoxity end-point assays. Interestingly, some types of chemotherapeutic drugs cause an increase in the impedance, while others decrease it. Another device, capable of assessing the resistance of spheroids, was developed as a combination of a planar organic electrochemical transistor (OECT) and a microfluidic trapping device (Curto et al., 2018). In this device, the spheroids made of epithelial cells forming tight junctions demonstrated much higher resistance than the spheroids that consisted of loosely connected fibroblasts.

Bürgel et al. constructed an automated multiplexed electrical IS (AMEIS) platform for the analysis of the spheroids in a microfluidic setting. This device, which obviated the need for pumps by utilizing a tilting stage (Bürgel et al., 2016), includes 15 separated capillaries connected to two reservoirs each, and a pair of measuring electrodes placed in the center of each capillary. When individual spheroids were manually injected into the chambers, and constant amplitude AC voltage applied between the electrodes, the passing of the spheroid between the electrodes lead to a drop in the current. The magnitude of this drop was proportional to the size of the spheroid. AMEIS devices are useful for quantifying cytotoxic effects seen in tumor cell spheroids treated with chemotherapeutic drugs, and, in slightly different settings, for the registration of action potential of the spheroids made of cardiomyocyte.

Thielecke et al. (2001b) placed the spheroids in a capillary based system for measuring their impedance with the aid of a precision pump. A comparison of spheroids made of butyrilcholinesterase knockdown cells and the controls showed that the former were smaller, contained a necrotic core, and had lower impedance in a wide range of frequencies (Thielecke et al., 2001b). Notably, simultaneous measurement of the impedance at low and high frequencies allows the determination of the volume fraction of cells comprising a spheroid. Subsequently a similar technique was successfully applied for the long term monitoring of the osteogenic differentiation of human mesenchymal stem cell cultures (Hildebrandt et al., 2010). For thorough review of impedance-based assays in stem cell cultures, we should refer to Gamal et al. (2018), who summarized achievement in this area recently.

A hanging drop platform has been IS-enabled by Schmid et al. (2016), who integrated an inlay with two pairs of platinum electrodes into the drop support structure. The distance between large electrodes placed within the drop radius was made as wide as possible (1.0 × 0.4 mm^2^), while remaining within the geometrical margins of the inlay, confining the electrical field in the conducting liquid. As a function of the drop volume and height, the electric field lines may either compress or expand, which changes the impedance between the electrodes, thus, enabling the measurement of the size of the hanging drop. To provide optical access, relatively small electrodes (0.5 × 0.2 mm^2^) were placed close to the presumed spheroid location, but off its center. Within the drop, the spheroids lift upwards, to the location between the electrodes. The presence of the spheroid disturbs the electric field lines and, consequently, changes the impedance. The measurements conducted in equidistant steps in the range of frequencies from 100 Hz to 40 MHz, helped to evaluate the relative sizes of the spheroid and the drop. In the case of spheroids made of cardiac cells, a set of specific frequencies was utilized to register its beating patterns.

3D cultures of cells embedded into the hydrogels or other porous scaffolds are a popular alternative to culturing cells as spheroids. Moreover, this approach is applicable for primary organoids. To date, several cases of IS application for 3D cultures of cells in gels were described. For example, Lin et al. constructed a perfusion culture system for real time monitoring of cell growth with a microelectrode array (Lin et al., 2009). In this system, cells grew within a 3D matrix synthesized from a polyethylene glycol hydrogel supplemented with poly-D-lysine *in situ*. Cell proliferation was measured by IS. Unfortunately, observed impedance kinetics of non-dividing neurons and fast proliferating fibroblasts were quite similar, thus, raising caution concerning the interpretation of the collected data.

Bagnaninchi et al. (2003, 2004; Bagnaninchi, 2010) grew the cells in microporous scaffolds and used an open-ended coaxial probe to measure their complex permittivity in the frequency range of 20 MHz−2 GHz. In these settings, the porosity of a scaffold and the cell concentrations were evaluated simultaneously (Bagnaninchi et al., 2003). Subsequently, this method was successfully used to assess variation in the morphology of the cells (Bagnaninchi et al., 2004; Bagnaninchi, 2010) and to discern normal and malignant variants of human lung cells embedded into low-conductive agarose hydrogels.

Using a pair of vertical electrodes, Lei et al. (2014) have performed rather complex IS measurements in 3D cell cultures grown in perfused agarose layers. For cell counting, the sensitivity of the technique peaked at the relatively low frequency of 500 Hz. When the same device had been employed in a real-time study of cytotoxicity, an increase rather than a decrease in impedance was observed, in sharp contrast to the data obtained in a planar electrode device constructed by the same group (Lei et al., 2012, 2015, 2017). In a colony formation assay, simultaneous quantification of cells (based on the absolute value of the impedance) and measuring colony size (based on measurements of the phase angle) was achieved. Subsequently, the same group of researchers employed impedimetric quantification of cells grown on a hydrogel-supporting paper substrate to construct a prototype for a high throughput screening of cancer cell chemosensitivity in point-of-care medical settings (Lei et al., 2016, 2018).

By inserting either three or four electrodes, Canali et al. (2015a,b) monitored spatial distribution of cells in larger 3D scaffolds. An exchange of working, counter and reference electrodes provided a variety of homogeneous electromagnetic field configurations and enabled coverage of every corner of the culturing chamber. The study revealed that cells tend to proliferate in the center of the culture chamber rather than in proximity of the chamber walls or in corners. Unfortunately, configuring electrode positions within the chamber requires multiple simulations, making the technique developed by Canali et al. far from easy to use.

More advanced application of impedance spectroscopy was developed to profile effects of various drugs on cancer cells embed in a 3D gel matrix (Pandya et al., 2017). This study was performed in a square chamber with interdigitated microelectrodes screening the frequencies ranging from 100 Hz to 1 MHz. After addition of a drug, observed magnitude of impedance of the drug-sensitive and drug-tolerant cancer cell cultures decreased over a period of 12 h. Notably, in sensitive cells, the drop in impedance was steeper than that in resistant ones, giving hope for utilization of this system for anticancer efficacy testing *in vitro*.

**Figure 9:**
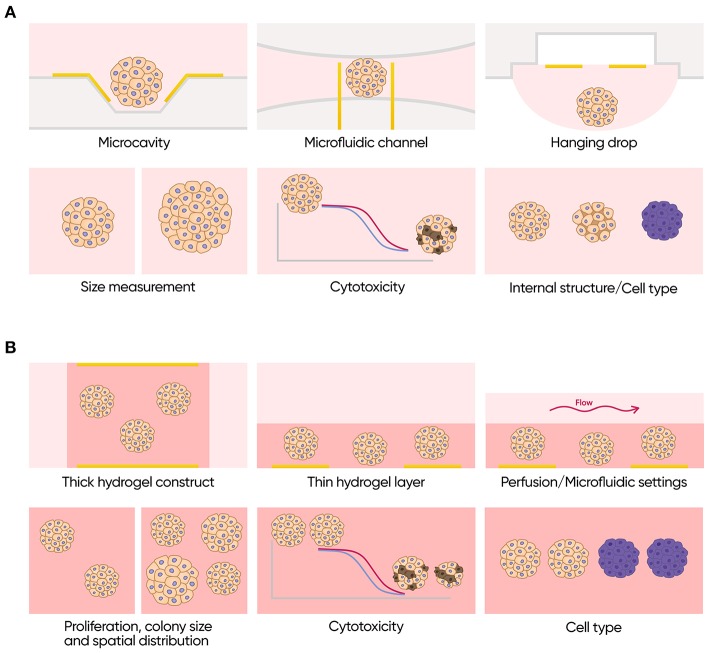
Applications of impedance spectroscopy for 3D cell models. **(A)** Isolated scaffold-free spheroids. Possible formats (top row): microcavity array, microfluidic channel with integrated electrodes and hanging drop array with integrated electrodes. Possible applications (bottom row): size measurements, cytotoxicity assay, and investigation of internal structure and cell type composition. **(B)** Scaffold based 3D cell culture models. Possible formats (top row): thick scaffold-based constructs with the electrodes placed on the opposite sides of it, thin gel matrix layers covered with cell culture media containing electrodes only on the bottom and perfused or microfluidics formats. Possible applications (bottom row): measurements of proliferation rate colony size and spatial distribution of the cells, cytotoxicity assay and investigation of cell type composition.

To date, IS has not been used for monitoring of primary 3D epithelial organoids. Existing data, however, suggest that this technique may be extremely useful for these models (Figure 9). Its proven ability to discriminate between different types of cells, to measure volume fraction of cells in a spheroid and its size, and to assess effects of different compounds in real time without any labels may significantly improve penetration of such models to both research and commercial testing fields. Moreover, application of impedance in combination with engineered epithelial tissues and organ-on-a-chip devices may lead to a construction of easily automatable physiologically relevant pipelines for drug development and for personalized medicine.

## Current State-of-the-art and Future Directions

Impedance measurements are already employed by cell biologists all over the world, especially those exploring barrier tissues in various 2D culture formats. Historically, an integrity of reconstructed barriers was probed by exposing them to detectable molecules, for example, fluorescent dye lucifer yellow or enzyme horseradish peroxidase, which are capable of penetrating these barriers exclusively through paracellular route (Hidalgo et al., 1989; Hubatsch et al., 2007). Data collected in this manner are reliable enough, and have, indeed, resulted in many important insights; working with cell layer permeating dyes is, however, quite laborious and time-consuming. Impedance measurement is an attractive alternative which has almost completely replaced label-based methods in routine quality control of *in vitro* models of barrier tissues. One of the most popular commercially available devices for the measurement of barrier function in academic settings is EVOM^2^ (World Precision Instruments), which allows small-scale experiments outside a cell culture incubator.

Next generation of the systems for *in vitro* models of barrier tissues was built for compatibility with the cell culture incubators. One example of that kind of systems is TEER24 (Applied Biophysics), which automatically collects impedance characteristics in real time on a single frequency of 75 Hz. Another, more advanced device cellZscope (nanoAnalytics GmbH) automatically profiles impedance over a broad range of frequencies, while also measuring electrical capacitance in real time. Recent works proved that measuring of electrical capacitance may be useful for evaluation of various 3D structures formed in process of growth and differentiation of cells *in vitro*. For example, in intestinal model, electrical capacitance gradually increases along with formation of villi form (van der Helm et al., 2019), while in course of the development of multilayer structure of epidermis it decreases (Groeber et al., 2015). Another study showed that the impedance increases linearly with an increase in extracellular deposition of collagen and hyaluronan, but changes in a more complex manner with incorporation of bone-specific compound hydroxyapatite (Kozhevnikov et al., 2019). A simple and rapid way to assess the state of either cellular or extracellular 3D structures opens up novel avenues for both quality control and for fundamental research.

One of the main disadvantages of existing automated systems for monitoring of the barrier function is their low throughput. All of them are designed to work with up to 24 membrane inserts which is definitely not enough for large-scale screenings. Today, 96-well plates with membrane inserts are commercially available; there is hope that impedance measuring systems compatible with this format should appear soon. Even if prototypes allowing microfluidic chip compatible monitoring of barrier function with IS have been repeatedly reported, no solutions of this kind were introduced to the market yet.

On the other hand, impedance measurements have been successfully employed for monitoring of 2D cultures of adherent cells growing directly on the electrodes (Giaever and Keese, 1984, 1986, 1991; Ke et al., 2011). To date, several systems including xCELLigence (ACEA Biosciences) and ZTheta (Applied Biophysics) were made available commercially for assessing proliferation rate, cell adhesion, migration and invasion as well as cytotoxicity of various compounds. Traditionally, assaying of cytotoxicity also relied on various labeled molecules (Riss et al., 2004), including MTT, MTS and ATP, with latter being based on firefly luciferase. All these methods have proved their worth in the labs, but almost all of them are end-point, and time-consuming. A few real time cytotoxicity assays do exist, with RealTime-Glo™ MT Cell Viability Assay (Promega) being probably the most popular one. This assay allows to record luminescent signal, which is proportional to the number of viable cells, over a period of a few days. Unfortunately, real-time versions of end-point assays require use of sophisticated plate reader, equipped with gas control unit capable to reconstruct the environmental conditions in a manner similar to cell culture incubators. In contrast, impedance-based assays allow continuous monitoring of cell cultures for sufficiently longer periods, and are built to fit incubators to begin with.

Real-time imaging systems such as IncuCyte S3 (Essen BioScience) certainly may be viewed as an alternative to impedance-based analysis. In this analysis, automatic bright field microscope equipped with several fluorescent channels is placed directly inside a standard cell culture incubator to provide real-time estimates of the proliferation and migration rates, cytotoxicity and many other parameters with an aid of different fluorescent dyes. Fluorescent microscopy based monitoring allows great flexibility for continuous study of cells in culture; however, IS based techniques outperform microscopy in ease of accessing resultant data and in its compatibility with high throughput screening platforms. As data obtained by IS and fluorescent microscopy complement each other, a hybrid RTCA eSight system (ACEA Biosciences), which combines automatic microscopy with impedance analyzer, has been recently launched to the market.

One more interesting extension of impedance measuring systems introduces additional sensors for monitoring of such important parameters as pH or oxygen content (Lei, 2014; Alexander et al., 2019). These sensors themselves may also be impedance-based as the surface of the electrodes can be functionalized with various molecules selectively recognizing one or another component of culture medium (Liu et al., 2018; Seo et al., 2018). Commercially available examples of these systems include microfluidic IMOLA-IVD (Cellasys), which allows measuring both pH and dissolved oxygen along with impedance of the cells. Therefore, combination of IS with other techniques significantly expands the capabilities of the method. Another important development is recently described combination of impedance flow cytometry and electric IS within the same microfluidic device suitable for single cell measurements allowing to evaluate properties of heterogeneous populations of cancer cells by dealing with them one at a time (Feng et al., 2019). It is expected that more of the hybrid cell analysis systems of this kind should be introduced in the future.

As 3D models of human tissues are getting popular and are expected to eventually replace 2D analogs, specific challenges of these models have to be taken into account. Only a few conventional assays were designed specifically for 3D cell models, for example, a 3D modification of end-point viability assay CellTiter-Glo (Promega) features a mix of reagents that penetrates large spheroids and has increased lytic capacity. Some automatic imaging systems, like IncuCyte S3 (Essen BioScience), allow reliable real-time monitoring if all the 3D spheroids lay on the same flat surface, which is not always the case. Moreover, the size of the spheroids is not always uniform, and not directly proportional to the number of its constituent living cells, which complicates interpretation of the data. These challenges call for alternative means of non-invasive, label-free longitudinal monitoring of cell states. Impedance spectroscopy is the method to meet these challenges. A particularly attractive feature of IS monitoring is real time measurement of both the parameters of the cells and the parameters of the culture medium and the extracellular matrix, thus, allowing scaling and standardization of continuous cell-based assays. A majority of reviewed 3D cell models have not been yet adopted as an industry standard. An integration of IS technique into these models may greatly facilitate the process.

Above we discussed some 3D culture based IS-enabled devices which primarily aim at cancer research, where they are believed to be instrumental in streamlining preclinical trials due to significantly better recapitulation of the tumor microenvironment. When seeded with a particular sample of primary cells reflecting underlining genetics of a certain individual, IS-enabled devices open up new horizons for personalized medicine. In the near future, IS technologies are expected to become a critical component of organotypic models suitable for high-throughput assaying which will eventually replace both laboratory animals and static *in vitro* cell models.

To summarize, the strongest and the most attractive features of impedance-based assays are their compatibility with high-throughput format and support for the measurements in real time with high temporal resolution. It is envisioned that most automated and the least labor-intensive assays of this and other kinds would be eventually accepted as the industry standard. So far, IS remains one of the very few techniques available for studying kinetics of a biological process rather than the resulting end points. As IS assesses biological properties based on electrical parameters of the system, an increase in sophistication of electrode layouts and experimental designs is expected, which will eventually lead to improvement of the precision and expansion of the palette of its applications. However, interpreters of IS-based data should proceed with caution, due to imperfect understanding of the parameters that have their effects on the impedance, especially in 3D cell models. Moreover, as the theory of IS stems from electromagnetic theory and is quite complex, work on popularization and explanation of the method for experimental biologists is required. Finally, we should mention one more factor preventing widespread of the impedance-based assays: the cost of consumables. Current situation should, however, improve with the rise in the popularity of IS techniques which would enable the large-scale production of the consumables.

## Author Contributions

TG and SN wrote the manuscript. GZ draw a part of the figures and edited the manuscript. AP, VP, AB, and AT edited the manuscript and contributed various ideas.

### Conflict of Interest

TG, SN, and VP are affiliated with SRC Bioclinicum; AT is affiliated with art photonics GmbH. The remaining authors declare that the research was conducted in the absence of any commercial or financial relationships that could be construed as a potential conflict of interest.
